# Surgery

**Published:** 1837-01

**Authors:** 


### SURGERY.

### *On the Treatment of Vascular Nævi by Injection.* By E. A. LLOYD, Esq.

IMPRESSED with the danger of attempting to cure large nævi by excision, or by caustic, or by the ligature, Mr. Lloyd was led, several years ago, to inject them with a stimulating liquor; and his success has answered his expectations. He uses a syringe with several sets of tubes adapted to the size of the nervus, and he introduces the point of the tube through an aperture in the skin at some little distance from the disease, as there is then greater facility in compressing the nævus so as to prevent hemorrhage. Before injecting it, the nævus should be emptied of blood by pressure, and the pressure kept up till the instant of injection. The fluid should be retained in the nævus from five to ten minutes, by making pressure along the track which had been occupied by the tube of the syringe. Very large nævi require to be injected at several places, but it is better to make one attempt on the first occasion. When very hard, they may require puncturing in various directions, but this should be done from a single point. It is better to make pressure round the nævus, to prevent extravasation of the injection in the cellular tissue; and this can easily be done with the cover of a pill box, a notch being cut in it for the admission of the point of the syringe. Mr. Lloyd has used different fluids: such as one part of strong nitric acid to ten or fifteen parts of the spirit of nitric ether; or the spiritus ammoniæ aromaticus, when others have failed; or solutions of chloride of lime, of sulphate and acetate of zinc, of muriate of ammonia, of hydriodate of potash, &c. Mr. Stanley has used wine with success. The chief advantages of this plan are, that it is applicable to nævi so large as to be wholly irremediable by other means, that it occasions no deformity, and produces very little pain or constitutional disturbance.

It is important to remember that the nævus in the first instance is generally very small, and that it should be at once attended to. At an early stage the nitric acid or potassa fusa are the best applications; and Mr. Lloyd is in the habit of applying a thick coating of sealing-wax varnish around the nævus to confine the action of the caustic to the disease itself. *Medical Gazette, October* 1, 1836.

---

### *On Anomalous Affections of the Larynx requiring Tracheotomy.* By W. H. PORTER, Surgeon to the Meath Hospital.

THIS very interesting memoir contains the detail of several cases operated on by the author, and in which the life of the patient was evidently saved, although the exact nature of the affection could not be ascertained. It is perfectly evident, Mr. Porter observes, "that an obstruction to the process of respiration may exist to a degree to *demand* the prompt interference of the surgeon, without our being able to calculate on its pathological exciting cause; and this obstruction may continue for a length of time, and the patient subsequently recover as completely as if such lesion of function had never existed; thereby proving that it was independent of any organic lesion of structure. At the same time it must be confessed, that the majority of subjects thus affected do *not* recover, and therefore in them a lesion of structure may be inferred, although, in the present state of our knowledge, we may not be able to pronounce accurately upon it."

In two of the cases described by Mr. Porter, an interesting physiological fact was demonstrated, viz. "that the integrity of the trachea is not necessary to the production of articulate sounds in the larynx." The subjects of these cases could speak very distinctly without closing the aperture in the trachea made by the operation.

*Dublin Journal for September,* 1836.

---

### *Case in which a Pebble remained eight weeks in the Larynx.* By HENRY BULLOCK, M.R.C.S.

THIS accident occurred in a child, six years of age, on the 21st of May. The pebble was of the size of a large horse-bean. At first there was a severe paroxysm of suffocation, which lasted half an hour; but subsequently the symptoms were mild, and so much resembled those of hooping-cough, that it was doubted if the pebble had been swallowed, although the child persisted in affirming this. The slight symptoms of bronchitis which supervened seemed subdued, and, from the 20th June to the period of her death, there was no return of cough; but, on the 6th of July, symptoms of pulmonic inflammation came on, and she died on the 18th. The pebble was found in the lower part of the larynx, surrounded with lymph: both the lungs and pleura were inflamed. *Med. Gazette, September* 17.

---

*On Extracting Foreign Bodies from the Ear.* By N. Hill, M.D., Greenock.

Dr. Hill has found the small hooked instrument which is to be found in cases of eye instruments useful in removing foreign bodies from the ear. This hook is so slender and light, having the point well turned up, that it can be used with all the delicacy of a probe, and can easily be carried along the parietes of the meatus, so as to get behind any object. Some of these cases are so troublesome, that any hint on the subject is worth attending to. *Edinburgh Journal, October,* 1836.

---

*On Lithotrity as practised by the late* Philip Fernandez, Esq.

This young surgeon, whose early death is much to be deplored, seems to have acquired a singular facility in seizing and crushing the stone in the bladder. The principle of his success is thus stated: "He saw that, when the bladder was injected, the surface would no longer be rugous but smooth, and that the stone would consequently be sure to roll to the lowest spot. He made it a rule in his operations, therefore, to come exceedingly close to the stone without touching it; and then, having first opened his instrument, he gently pressed the part. The spot touched being thus made the lowest part of the bladder, the stone uniformly rolled into the instrument, and he had nothing to do but to close it and crush the calculus."

*Medical Gazette, October* 22.

---

*Aneurism of the Arteria Innominata successfully treated by Ligature of the Common Carotid.* By S. W. Fearn, Esq. Surgeon, Derby.

It is unnecessary to give the details of this case, which is creditable to the operator. As the author justly remarks, "it tends strongly, with the cases already published, to determine *one* important surgical question, viz. the curableness of what has till lately been considered incurable, aneurism of the arteria innominata; and it also bears upon the general question of the propriety, in every case, of attempting the cure of aneurism by ligature of the vessel on the distal side of the disease." *Lancet, October* 15.

---

*On Amputation of the Penis.* By J. Morrison, M.D., Newry.

Dr. Morrison relates two cases of amputation of the penis, from which he is inclined to prefer removing the organ with one stroke of the knife, instead of the circular incision usually recommended; for, even by the former method, which is less painful, enough skin is preserved to cover the wound. He recommends the introduction after the operation of a quill canula, two inches long, instead of a catheter, which irritates the neck of the bladder. The canula gives free exit to the urine, prevents an undue contraction of the cut urethra, and is unirritating. Sutures are preferable to adhesive straps. *Dublin Journal, September,* 1836.

---

*New Treatment of Onychia Maligna.* By C. Ray, Esq., Falkingham.

Mr. Ray is opposed to the entire evulsion of the nail in this most troublesome disease, and justly states that it is useless to expect a cure until that part of the nail, at least, which acts as a constant source of irritation is removed. His mode of treatment is, after freely dividing the swollen parts over the root of the nail and cauterising them, to apply poultices, and enjoin perfect quiescence of the limb. This mode of treatment, the caustic being reapplied every second or third day, brings the parts into a state favorable for the partial removal of the nail, which is effected by passing a scissors from within outwards beneath the posterior angles on each side, and removing "an exact triangular portion," the two incisions meeting in a point in the centre of the posterior edge of the nail. After the separation of the nail, the wound is dressed with adhesive straps which are not to be removed for some days.

*Lancet, November* 12, 1836.

